# Smart protein biogate as a mediator to regulate competitive host-guest interaction for sensitive ratiometric electrochemical assay of prion

**DOI:** 10.1038/srep16015

**Published:** 2015-11-04

**Authors:** Peng Yu, Xiaohua Zhang, Jiawan Zhou, Erhu Xiong, Xiaoyu Li, Jinhua Chen

**Affiliations:** 1 State Key Laboratory of Chemo/Biosensing and Chemometrics, College of Chemistry and Chemical Engineering, Hunan University, Changsha, 410082, P.R. China

## Abstract

A novel competitive host-guest strategy regulated by protein biogate was developed for sensitive and selective analysis of prion protein. The methylene blue (MB)-tagged prion aptamer (MB-Apt) was introduced to the multiwalled carbon nanotubes-β-cyclodextrins (MWCNTs-β-CD) composites-modified glassy carbon (GC) electrode through the host-guest interaction between β-CD and MB. In the absence of prion, MB-Apt could be displaced by ferrocenecarboxylic acid (FCA) due to its stronger binding affinity to β-CD, resulting in a large oxidation peak of FCA. However, in the presence of prion, the specific prion-aptamer interaction drove the formation of protein biogate to seal the cavity of β-CD, which hindered the guest displacement of MB by FCA and resulted in the oxidation peak current of MB (*I*_MB_) increased and that of FCA (*I*_FCA_) decreased. The developed aptasensor showed good response towards the target (prion protein) with a low detection limit of 160 fM. By changing the specific aptamers, this strategy could be easily extended to detect other proteins, showing promising potential for extensive applications in bioanalysis.

Prion protein is mainly located in the neuronal cells of the central nervous system in mammals, which is thought to be involved in the pathogenesis of transmissible spongiform encephalopathies, such as Creutzfeldt-Jakob disease in humans, bovine spongiform encephalopathy in cattle, and scrapie in sheep^1^,^2^. According to the protein-only hypothesis, conformational change from normal cellular form (PrP^C^) into its infectious isoform (PrP^Sc^) is a crucial step in prion propagation^3^. Therefore, it is very important to detect the concentration of prion in blood. Immunological techniques (Western blot and enzyme-linked immunosorbent assays) are one of the most accurate methods for the detection of prion^4^. However, these methods are time-consuming^5^,^6^, and are cumbered for widespread use of rather expensive antibody-enzyme conjugates^7^. Furthermore, due to the very low concentration of PrP^Sc^ in blood (down to picomolar), the accurate detection of prion is difficult. Therefore, the development of simple, inexpensive and sensitive method for the prion assay is highly desirable for early diagnostics of prion diseases.

Compared with traditional molecular recognition system, aptamers (single stranded DNA or RNA molecules selected from random-sequence nucleic acid libraries using systematic evolution of ligands with exponential enrichment (SELEX)^8^,^9^) exhibit many advantages, including simple synthesis, easy labeling, good stability, and wide applicability^10^,^11^,^12^. Due to their distinctive properties, aptamers have been widely employed as the recognition elements to construct biosensors^13^,^14^,^15^,^16^,^17^. Among many kinds of aptamer-based biosensors, electrochemical aptamer-based (E-AB) biosensors have received great interests because of the simplicity, fast response, relatively low cost and low power requirement of electrochemical methods^18^,^19^. However, up to now, only a few works addressed on the prion detection based on the E-AB biosensors^20^,^21^,^22^. Korri-Youssoufi *et al.* developed an E-AB biosensor for prion assay based on “signal-off” strategy^21^. The electrochemical signal in his work was detected by ferrocenyl group incorporated between dendrimers and aptamers layers, thus developing a new platform for connecting redox markers and aptamers. However, such “signal-off” sensor suffers from limited signaling capacity, in which only a maximum of 100% signal suppression can be attained under any experimental conditions^23^.

Recently, ratiometric assays, including ratiometric electrochemical^24^,^25^,^26^,^27^,^28^ and ratiometric electrochemiluminescent^29^,^30^ methods, have attracted growing attentions. The ratiometric electrochemiluminescent assay, based on dual-potential electrochemiluminescence (ECL) measurement, needs to create ECL report units with two emitting states that have the potential-dependent properties upon the substrate concentration, while the ratiometric electrochemical assay needs to choose two redox probes which can be electrochemically oxidized/reduced at different potentials. Both of them possess good analytical performances (such as low detection limit, good reliability and reproducibility), which inspires us to construct the ratiometric sensing platform for prion assay.

On the other hand, cyclodextrins (CD) are oligosaccharides composed of six, seven, or eight glucose units (α-, β-, or γ-CD, respectively), which are toroidal in shape with a hydrophobic inner cavity for specific recognition of guest molecules and a hydrophilic exterior to provide water solubility^31^,^32^. Due to their high recognition of guest molecules, good water-solubility, well biocompatibility and environmental friendliness, several electrochemical biosensors have been developed on the basis of host-guest recognition technique to detect biologically relevant analytes in recent years^33^,^34^,^35^,^36^,^37^. However, all of them are based on the single-signaling strategy. To the best of our knowledge, the application of the competitive host-guest interaction in ratiometric E-AB biosensors based on the dual-signaling strategy has not been reported.

In this paper, we designed a protein biogate to regulate the host-guest competitive interaction between β-CD and two reporting probes and successfully applied such strategy to the fabrication of ratiometric electrochemical aptasensor for selective and sensitive detection of prion. Here, One of the probes was methylene blue (MB) attached to one end of the prion aptamer, and the other was ferrocenecarboxylic acid (FCA). Although both MB and FCA could form the inclusion complex with β-CD, FCA was used as a competitive guest for its higher hydrophobic property. As shown in Fig. 1, the multiwalled carbon nanotubes (MWCNTs) and β-CD composites were used to modify the glass carbon (GC) electrode and β-CD worked as the carrier to bring the prion aptamer to the electrode through the host-guest interaction between β-CD and MB. The introduction of MWCNTs here was beneficial to enhancing the electron transfer of the electrochemical sensor and providing a high surface-to-volume ratio. Due to the specific interaction between the MB-tagged aptamer and prion protein, the cavity of β-CD was sealed by prion protein, causing the impediment to the displacement of MB by FCA. The prion protein functioned as a biogate to regulate the competitive host-guest interaction between β-CD and the redox probes. However, other proteins could not interact with MB-tagged prion aptamer (MB-Apt), resulting in the unsuccessful formation of protein biogate and thus the displacement of MB by FCA. Based on the ratio between the oxidation peak current of MB (*I*_MB_) and that of FCA (*I*_FCA_), prion was detected sensitively and selectively. In comparison with other reported ratiometric electrochemical biosensors that usually needed one multi-labeled DNA strand or at least two single-labeled DNA strands^24^,^25^,^26^,^27^,^28^, we just used only one single-labeled target aptamer to construct the ratiometric electrochemical aptasensor, which can potentially lower the cost and simplify the ratiometric E-AB sensor. Moreover, this strategy could have promising application in the detection of a wide range of analytes by changing the corresponding specific aptamers.

## Results

### Characterization of the developed aptasensor and Feasibility of the ratiometric E-AB sensor for prion assay

**Figure 2:**
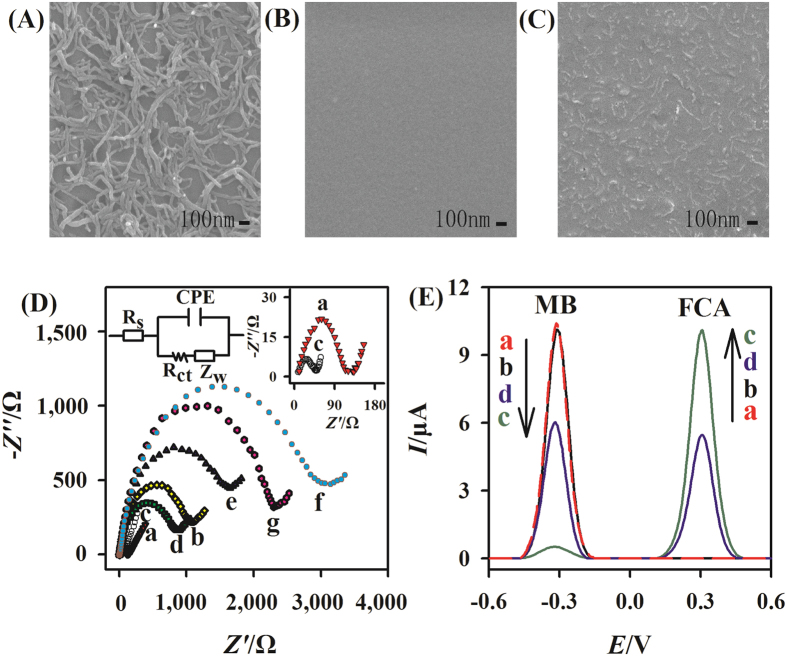
Characterization and Feasibility of the developed aptasensor for prion assay. SEM images: (**A**) MWCNTs, (**B**) β-CD, (**C**) MWCNTs-β-CD composites. (**D**) Electrochemical impedance spectra (Nyquist plots) of the different modified electrodes in 0.1 M KCl aqueous solution containing 5 mM (1:1) [Fe(CN)_6_]^3–/4–^. (a) Bare GC electrode, (b) β-CD/GC electrode, (c) MWCNTs-β-CD/GC electrode, (d) MB-Apt/MWCNTs-β-CD/GC electrode, (e) BSA/MB-Apt/MWCNTs-β-CD/GC electrode, (f) prion/BSA/MB-Apt/MWCNTs-β-CD/GC electrode, and (g) FCA/prion/BSA/MB-Apt/MWCNTs-β-CD/GC electrode. Inset: equivalent circuit model for electrochemical impedance measurement system (the upper left corner) and the nyquist plots of the bare GC electrode and MWCNTs-β-CD/GC electrode (the upper right corner). (**E**) SWV curves of the different electrodes. (a) BSA/MB-Apt/MWCNTs-β-CD/GC electrode, (b) BSA/MB-Apt/MWCNTs-β-CD/GC electrode incubated with 200 nM prion, (c) BSA/MB-Apt/MWCNTs-β-CD/GC electrode incubated with 20 μM FCA without prion, (d) BSA/MB-Apt/MWCNTs-β-CD/GC electrode incubated with 1 pM prion and then 20 μM FCA.

Firstly, the morphologies of acidified MWCNTs, β-CD, and MWCNTs-β-CD composites were characterized by SEM. It is noted that the morphology of the MWCNTs-β-CD composites (Fig. 2C) is much different from that of acidified MWCNTs (Fig. 2A) and β-CD (Fig. 2B), and acidified MWCNTs are dispersed highly in the MWCNTs-β-CD composites. Then, the EIS method was used to study the interface properties of the modified electrodes. Figure 2D shows the electrochemical impedance results of the different electrodes. Based on the charge transfer kinetics of [Fe(CN)_6_]^3–/4–^ redox probe, faradic impedance spectra can be modeled with the Randles’s equivalent circuit approach^38^, as shown in the inset (in the upper left corner) of Fig. 2D. The circuit includes a commonly used electrolyte resistance (Rs), constant phase element (CPE), Warburg impedance (Zw) and charge-transfer resistance (Rct). Among them, Rct is the most directive and sensitive parameter in response to the change at the electrode/solution interface^39^,^40^. It is noted that the bare GC electrode shows a very small semicircle domain (Rct = 124 Ω, curve a in Fig. 2D), indicating a very fast charge-transfer process of [Fe(CN)_6_]^3–/4–^ ions. However, after the modification of the GC electrode with 2 wt.% β-CD solution, the β-CD film on the electrode surface, due to its poor electrical conductivity, effectively hinders the electron transfer and thus leads to an enhanced charge-transfer resistance (Rct = 994 Ω, curve b in Fig. 2D). In order to accelerate the electron transfer and improve the conductivity of the electrode, the MWCNTs-β-CD composites were used to modify the GC electrode to construct the aptasensor. Compared with the β-CD modified GC electrode, the Rct value of the MWCNTs-β-CD/GC electrode (58 Ω, curve c in Fig. 2D) is much smaller. Then, the self-assembly of the negatively charged MB-Apt causes a large increase in the Rct value (765 Ω, curve d in Fig. 2D), indicating that MB-Apt is successfully attached to the electrode through the host-guest interaction between β-CD and MB. The value of Rct further increases to 1673 Ω by blocking the electrode with BSA (curve e in Fig. 2D). After the specific interaction of prion (200 nM) with its aptamer (MB-Apt), it is noted that the value of Rct increases to 2763 Ω (curve f in Fig. 2D) due to the steric effect of prion protein-aptamer complex, suggesting a successful formation of the protein biogate on the electrode surface. After the further incubation with 20 μM FCA solution, the value of Rct decreases to 2294 Ω (curve g in Fig. 2D). This result indicates that some MB-Apt strands, which didn’t interact with prion protein, are successfully displaced by FCA and detached from the electrode surface. The above EIS results indicate that all the steps shown in Scheme 1 were successfully performed.

The feasibility of the developed aptasensor for prion assay is investigated by square wave voltammetry (SWV) method and the results are shown in Fig. 2E. It is noted that a high oxidation peak of MB at about −0.31 V is observed for the BSA/MB-Apt/MWCNTs-β-CD/GC electrode (curve a in Fig. 2E), indicating that the MB-Apt is successfully immobilized on the GC electrode through the host-guest interaction between β-CD and MB. When the BSA/MB-Apt/MWCNTs-β-CD/GC electrode is incubated with 200 nM prion, no obvious change of the oxidation peak current of MB is observed (curve b in Fig. 2E). This indicates that prion protein attached on the electrode has little effect on the electron transfer between the reporter (MB) and electrode. The possible reason is that the reporter is trapped in the cavity of β-CD and very close to the electrode surface. After the BSA/MB-Apt/MWCNTs-β-CD/GC electrode is incubated with 20 μM FCA without prion, the oxidation peak current of MB decreases significantly while a well-defined peak of FCA appears at about 0.30 V (curve c in Fig. 2E), due to the successful displacement of MB by FCA. When the BSA/MB-Apt/MWCNTs-β-CD/GC electrode is incubated with 1 pM prion and subsequently 20 μM FCA, a decrease of MB signal and an increase of FCA signal are observed (curve d in Fig. 2E) compared to curve a. This suggests that the protein biogate is successfully fabricated for prion assay and functions as a mediator to regulate the displacement of MB by FCA. Based on the results observed in Fig. 2E, it is no doubt that the developed aptasensor can be used for prion assay.

### Assay performance

**Figure 3:**
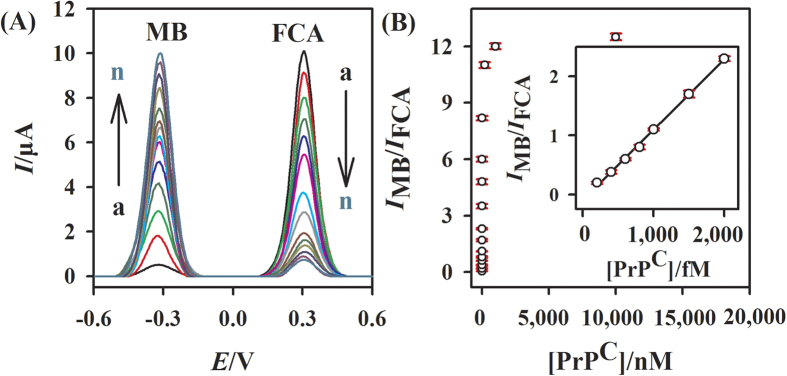
Sensitive detection of prion. (**A**) SWV signal changes of the developed ratiometric electrochemical aptasensor in the presence of different concentrations of prion. The concentrations are (from a to n) 0 fM, 200 fM, 400 fM, 600 fM, 800 fM, 1 pM, 1.5 pM, 2 pM, 10 pM, 100 pM, 1 nM, 10 nM, 200 nM, 10 μM. (**B**) Dependence of *I*_MB_/*I*_FCA_ on different concentrations of target protein. The inset shows linear calibration of *I*_MB_/*I*_FCA_ vs prion concentration. Error bars represent the standard deviations of three parallel experiments.

The developed ratiometric E-AB sensor was used for the detection of prion protein. As shown in Fig. 3A, it is noted clearly that the oxidation peak current of MB increases and that of FCA decreases with the increase of the concentration of prion. Figure 3B reveals that the value of *I*_MB_/*I*_FCA_ also increases with the increase of prion concentration (up to 1 μM) and then reaches a platform. In the range from 200 to 2000 fM, the value of *I*_MB_/*I*_FCA_ is a good linear fit to the concentration of prion, and the correlation coefficient (R^2^) is 0.9951. The limit of detection (LOD) at 3σ is 160 fM. Furthermore, as shown in Table 1, the LOD result obtained by the developed ratiometric electrochemical aptasensor is much lower than that reported in the literatures, indicating a good performance of the developed aptasensor towards the prion assay.

### Selectivity and reproducibility

**Figure 4:**
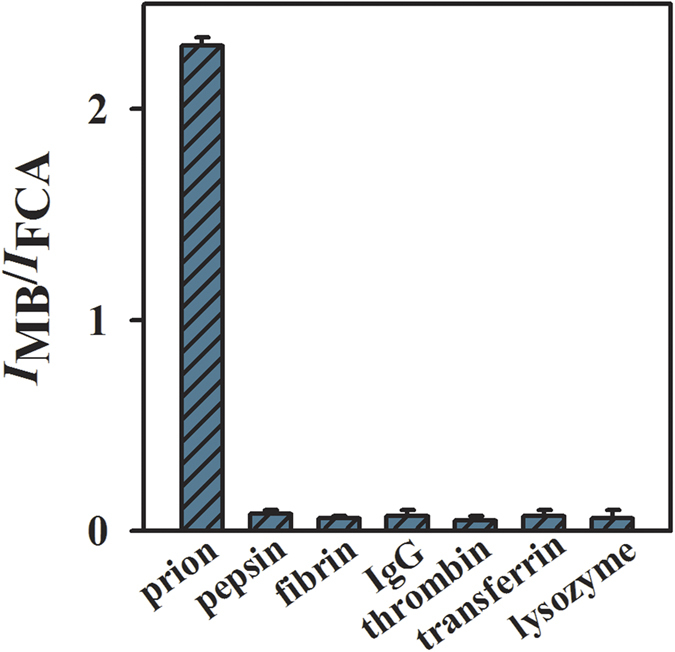
Selectivity of the biosensor to prion, pepsin, fibrin, IgG, thrombin, transferrin, lysozyme. Concentration of prion is 2 pM and the others are 2 μM. Error bars show the standard deviations of three parallel experiments.

In order to investigate the selectivity of the proposed aptasensor, control experiments were performed by using pepsin, fibrin, IgG, thrombin, transferrin, lysozyme as the interferents. The BSA/MB-Apt/MWCNTs-β-CD/GC electrode was incubated with the interferents and prion (2 pM). As shown in Fig. 4, the prion sample generates predominant ratiometic readout, while other potential interferents induce negligible responses. It indicates that the proposed biosensing system exhibits an excellent selectivity for prion assay. The reproducibility of the BSA/MB-Apt/MWCNTs-β-CD/GC electrode was also investigated. Five modified electrodes were used to detect prion (1 pM) and the relative standard deviation is 1.4%, which suggest that the developed method has potential for further developments.

### Recovery test

**Figure 5:**
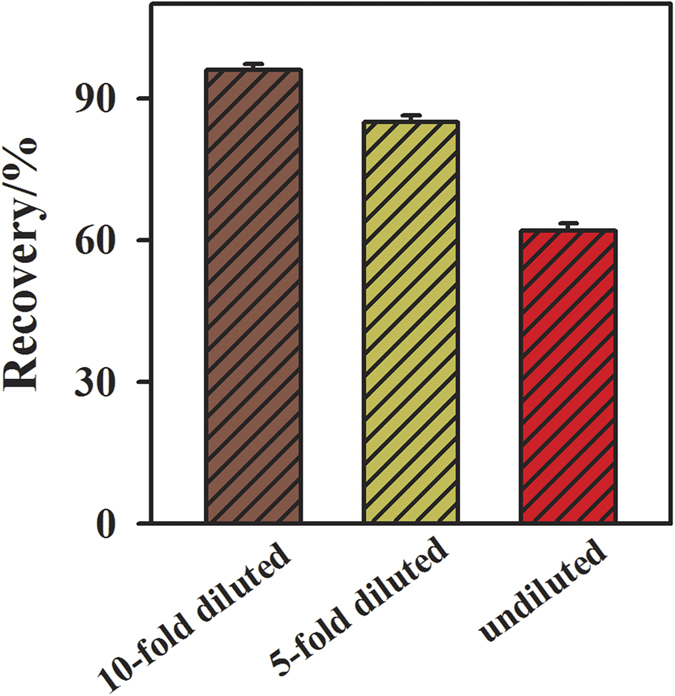
Effect of serum on the recovery test of prion. Serum was used without dilution or after dilution with 50 mM Tris, pH 7.4, and 50 mM NaCl, either 5-fold or 10-fold. The prion solution with 1 pM for recovery test was prepared either in undiluted serum or in the diluted serum. Error bars show the standard deviations of three parallel experiments.

For validating the practical application of the aptasensor, it is very significant and essential to detect prion in complex system. To do this, normal human blood serum samples were employed as the model of complex systems to evaluate the analytical applicability of the developed method. We compared the recovery results in the undiluted, five-fold diluted and ten-fold diluted serum samples. The results show that the highest rate of recovery for the added prion (1 pM) is obtained in the ten-fold diluted serum (Fig. 5). Although BSA was used as the blocking agent in this work, the undiluted serum does influence the sensing signal and hinders the interaction between prion and its aptamer to some extent, probably owing to the very high overall concentration of proteins and/or electrolytes in undiluted serum^41^,^42^. Hence, ten-fold diluted serum was used for the recovery test in this paper. At first, the aptasensor was incubated with the diluted human serum without PrP^C^. After incubation in this serum sample, according to the ratiometric response of the sensor measured, a reference concentration (C_0_) was calculated through the calibration equation obtained from Fig. 3B. Then the sensor response was measured at certain PrP^C^ concentrations (C_added_) of spiked serum, and the concentration of the PrP^C^ (C_1_) was also calculated through the calibration equation in the similar manner. The concentration of prion found (C_found_) was calculated (C_found_ = C_1_–C_0_). The value of C_found_/C_added_ has been multiplied by 100 to obtain the recovery in %. The recovery for the added prion with 0.5 pM, 1 pM, 1.5 pM, 2 pM are 94%, 96%, 98%, 95%, respectively. It is noted that the recoveries for all concentrations of prion are less than 100%. This may result from that the high overall concentration of proteins and/or electrolytes in serum hinders the interaction between prion and its aptamer to some extent^41^,^42^, as observed in Fig. 5. These results indicate that the recovery of the developed method is satisfactory and has great practical applications in prion assay.

## Discussion

By introducing protein biogate to regulate the competitive host-guest interaction between β-CD and redox probes, we developed a novel ratiometric E-AB sensor for sensitive and selective detection of prion protein. It has good analytical performance with a low LOD (160 fM), satisfactory selectivity and good reproducibility. Compared with the previously reported prion biosensors, the ratiometric electrochemical sensing strategy developed in this paper is more sensitive. We believe that our new analytical method will have promising applications in the sensitive and selective electrochemical determination of other proteins. And more importantly, such a design concept could improve the selectivity of the CD-based chemo/biosensors and widen the applications of CD in bioanalysis.

## Methods

### Materials

The recombinant human cellular prion protein, PrP^C^ (Human PrP^C^ (90–231), molecular weight 18.5 kDa) was supplied by Jena Bioscience (Jena, Germany). Based on the basic sequence of aptamer for PrP^C^ (90–231) selected by Takemura *et al.*^43^, the MB-tagged aptamer (MB-Apt) specific for PrP^C^ (90–231) with the dT_10_ spacer at the 5′ end was designed and then synthesized by Sangon Inc. (Shanghai, China). Its sequence is 5′-MB-dT_10_ GTT TTG TTA CAG TTC GTT TCT TTT CCC TGT CTT GTT TTG TTG TCT-3′. MWCNTs (purity > 95%, diameter 20–40 nm and length 5–15 μm) were purchased from Shenzhen Nanotech Port Co. (Shenzhen, China). The normal human serum used in the recovery test was supplied by Anyan Inc. (Shanghai, China). All other chemicals were of analytical grade. All the aqueous solutions were prepared using ultrapure water with an electrical resistivity of 18.2 MΩ cm obtained from a Milli-Q water purification system (Millipore Corp., Bedford, MA).

### Apparatus

All electrochemical measurements, including electrochemical impedance spectroscopy (EIS) and square wave voltammetry (SWV), were performed on a CHI 660B Electrochemical Workstation (Chenhua Instrument Company of Shanghai, China). A conventional three-electrode system was used with a glassy carbon (GC) electrode (3 mm in diameter) as the working electrode, a platinum wire as the counter electrode and a saturated calomel electrode (SCE) as the reference electrode. The morphologies of acidified MWCNTs, β-CD, and MWCNTs-β-CD composites were investigated by field emission scanning electron microscopy (SEM, JSM 6700, Japan).

### Preparation of the sensing interface and electrochemical detection of prion

Before use, MWCNTs were treated in a mixed acid solution (conc. H_2_SO_4_ and conc. HNO_3_, 3:1 V/V) under sonication for 3 h, then collected by centrifuge (10000 rpm, 20 min) and washed with ultrapure water several times^21^. The composites of MWCNTs and β-CD were prepared as follows: in brief, the obtained acidified-MWCNTs (1 mg) was added into a β-CD solution (2 ml, 2 wt.%) and the mixture was then ultrasonicated for 2 h to produce a black homogeneous ink (i.e., the MWCNTs-β-CD ink). Prior to use, the GC electrode was firstly polished with 0.5 μm and 0.05 μm alumina slurries until a mirror-like surface was obtained, followed by successive ultrasonic cleaning in ultrapure water, ethanol and ultrapure water to remove the residual Al_2_O_3_ powder. To obtain the MWCNTs-β-CD modified electrode (MWCNTs-β-CD/GC), 8 μL of the MWCNTs-β-CD ink was coated onto the GC electrode and dried in air. Then, MB-Apt (10 μL, 10 μM) was dropped onto the electrode for 1 h to form the MB-β-CD complex with the stability constant of 4.4 × 10^3^ M^−1^ due to the host-guest interaction between β-CD and MB^44^. The obtained electrode was labelled as MB-Apt/MWCNTs-β-CD/GC electrode. The obtained MB-Apt/MWCNTs-β-CD/GC electrode was further incubated with 1% (wt/vol) bovine serum albumin (BSA) for 1 h to avoid the nonspecific absorption. The obtained modified electrode was denoted as BSA/MB-Apt/MWCNTs-β-CD/GC electrode. For electrochemical detection of prion, the resulting BSA/MB-Apt/MWCNTs-β-CD/GC electrode was incubated with various concentrations of prion for 1 h to form the prion/BSA/MB-Apt/MWCNTs-β-CD/GC electrode. Subsequently, 10 μL of 20 μM FCA probe in buffer (34 mM Tris-HCl, 300 mM NaCl, 8.5 mM KCl, 5 mM MgCl_2_ and 1.7 mM CaCl_2_, pH 7.4) was dropped onto the surface of the prion/BSA/MB-Apt/ MWCNTs-β-CD/GC electrode and kept at room temperature for 1 h to obtain the FCA/prion/BSA/MB-Apt/MWCNTs-β-CD/GC electrode. After each step, the electrode was rinsed thoroughly with washing buffer (10 mM Tris−HCl, pH 7.4) and dried under a stream of nitrogen. Finally, the FCA/prion/BSA/MB-Apt/MWCNTs-β-CD/GC electrode was immersed in a buffer solution (100 mM PBS containing 100 mM KCl, pH 7.0) and investigated using SWV with a step potential of 4 mV, a frequency of 25 Hz, and an amplitude of 25 mV in the potential range between −0.6 and 0.6 V. Each measurement was repeated at least three times. The EIS measurements were performed to monitor each preparation step of the sensing interface in 0.1 M KCl aqueous solution with 5 mM [Fe(CN)_6_]^3−/4−^ as the probe. For comparison, the control experiments for pepsin, fibrin, IgG, thrombin, transferrin, lysozyme were carried out under the same conditions.

## Additional Information

**How to cite this article**: Yu, P. *et al.* Smart protein biogate as a mediator to regulate competitive host-guest interaction for sensitive ratiometric electrochemical assay of prion. *Sci. Rep.* **5**, 16015; doi: 10.1038/srep16015 (2015).

## Figures and Tables

**Figure 1:**
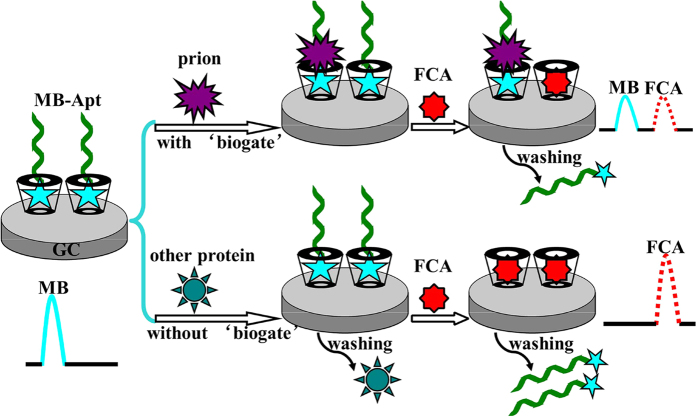
Schematic illustration of the ratiometric E-AB sensor for prion detection. The assay uses the target protein (prion protein) as a mediator to regulate the competitive host-guest interaction between β-CD and the redox probes. However, other proteins could not interact with MB-tagged prion aptamer (MB-Apt), resulting in the unsuccessful formation of protein biogate and thus the displacement of MB by FCA. Based on the ratio between the oxidation peak current of MB (*I*_MB_) and that of FCA (*I*_FCA_), prion was detected sensitively and selectively.

**Table 1:** Comparison of various methods for prion assay.

Method	Sensing element	LOD (pM)	Refs
Surface plasmon resonance	Aptamer	4000	[45]
Surface plasmon resonance	Monoclonal antibdies, in HEPES	570	[46]
Fluorescence	Quantum dots labeled aptamer	85.5	[47]
Electrochemical quartz crystal microbalance	Aptamer	50	[22]
Electrochemical quartz crystal microbalance	Antibody PRI 308	48	[22]
Electrochemical quartz crystal microbalance	Antibody BAR 223	20	[22]
Fluorescence	tetramethyl–6-carboxyrhodamine-aptamer	20	[41]
Differential pulse voltammetry	Ferrocene labeled aptamer	0.8	[20]
Cyclic voltammetry	Ferrocene labeled aptamer	0.5	[21]
SWV	MB labeled aptamer and FCA	0.16	This work
